# Perioperative coronary artery spasms in patients undergoing catheter ablation of atrial fibrillation

**DOI:** 10.1007/s10840-021-01089-6

**Published:** 2021-11-12

**Authors:** Masato Hachisuka, Yuhi Fujimoto, Eiichiro Oka, Hiroshi Hayashi, Teppei Yamamoto, Hiroshige Murata, Kenji Yodogawa, Yu-ki Iwasaki, Meiso Hayashi, Yasushi Miyauchi, Wataru Shimizu

**Affiliations:** 1 Department of Cardiovascular Medicine, Nippon Medical School, 1-1-5 Sendagi, Bunkyo-ku, Tokyo, 113-8603 Japan; 2 Mabori Medical Clinic, Yokosuka, Japan

**Keywords:** Atrial fibrillation; ST-T elevation, Coronary artery spasm, Catheter ablation, Complication

## Abstract

**Purpose:**

Catheter ablation (CA) is an established treatment for atrial fibrillation (AF). Although coronary artery spasms (CAS) during or after ablation procedures have been described as a rare complication in some case reports, the incidence and characteristics of this complication have not been fully elucidated. The present observational study aimed to clarify the CAS in a large number of patients experiencing AF ablation.

**Methods:**

A total of 2913 consecutive patients (male: 78%, mean 66 ± 10 years) who underwent catheter ablation of AF were enrolled.

**Results:**

Nine patients (0.31%, mean 66 ± 10 years, 7 males) had transient ST-T elevation (STE). Eight out of the 9 patients had STE in the inferior leads. STE occurred after the transseptal puncture in 7 patients, after the sheath was pulled out of the left atrium in 1, and 2 h after the ablation procedure in 1. Six patients had definite angiographic CAS without any sign of an air embolization on the emergent coronary angiography. In the3 other patients, the STE improved either directly after an infusion of nitroglycerin or spontaneously before the CAG. The patients with CAS had a higher frequency of a smoking habit (89% vs. 53%; *P* = .04), smaller left atrial diameter (36 ± 6 vs. 40 ± 7; *P* = .07), and lower CHADS_2_ score (0.6 ± 0.5 vs. 1.3 ± 1.1; *P* = .004) than those without.

**Conclusions:**

Although the incidence was rare (0.31%), CAS should be kept in mind as a potentially life-threatening complication throughout an AF ablation procedure especially performed under conscious sedation.

## Introduction

Atrial fibrillation (AF) is the most common cardiac arrhythmia, and radiofrequency catheter ablation (RFCA) for a pulmonary vein isolation is a well-established therapy for AF [1]. Although AF ablation is generally considered to be safe, serious complications can occur, some being fatal. Coronary artery spasms (CAS) during a perioperative period of AF ablation are rare, but potentially result in cardiogenic shock or ventricular fibrillation requiring cardiopulmonary resuscitation [2].

The past literature [3, 4] has suggested that CAS are more common in Japan than in the Western countries, but there have been some case reports [5, 6] of CAS during or around the AF ablation procedure in non-Asian patients. As the details of this complication have not been fully elucidated, this study was conducted to demonstrate the incidence, characteristics, and risk factors of CAS during the perioperative period of the ablation of AF.

## Methods

### Study subjects

This study retrospectively enrolled patients who underwent catheter ablation of AF at Nippon Medical School Teaching Hospital between August 2008 and October 2020. The study protocol was approved by the institutional review board of the hospital. Patients were excluded if they were < 20 years old or were diagnosed with CAS before the procedure. The patients that underwent surgical ablation procedures were excluded. Cases with a diagnosis of air embolism or with a high probability of air embolism were excluded.

### Ablation procedures

The intracardiac electrograms and surface electrocardiograms (ECG) were continuously monitored by two or more doctors performing the ablation, nurses assisting in the patient care and procedures, and clinical engineers operating the EP-recording system, stimulator, generator, and 3-dimensional (3D) Work Mate system (St. Jude Medical, Inc., St. Paul, MN, USA). A transseptal puncture was performed using the non-RF (BRK, St. Jude Medical) or RF needle (Baylis Medical, Montreal, Quebec, Canada) inserted through a long sheath (SL 0 and/or SL 8.5, St Jude Medical). In principle, after one septal puncture, the same site was passed by a guidewire, and one or two sheaths were used to approach the left atrium in addition to the ablation catheter. After the transseptal access, mapping catheters and an irrigated ablation catheter were inserted into the left atrium. A steerable sheath (Agilis, St. Jude Medical) was used for the ablation catheter in all procedures. The procedures were guided with an electroanatomical mapping system (CARTO, Biosense Webster, Irvine, CA, USA, or Ensite NavX, St. Jude Medical). The standard AF ablation strategy in our hospital was a circumferential pulmonary vein isolation with the endpoint of a complete isolation of all four pulmonary veins (PV). The creation of additional lesions depended on the discretion of the operator. Ablation of the ganglionated plexi was not attempted in any patients.

### Sedative agents

After informed consent was obtained from the patients, catheter ablation was performed under conscious sedation using intravenous midazolam (0.1–0.15 mg/kg) followed by a continuous intravenous administration of propofol at 0.3–3.0 mg/kg/h or dexmedetomidine at 0.2–0.7 µg/kg/h. Bispectral index (BIS) monitoring (Vista A-3000, Covidien Japan, Tokyo, Japan) [7] was used during the procedure, and the dosage of the sedative agents was adjusted to maintain the BIS within a range of 50–70.

### Transthoracic echocardiographic analysis

The patients underwent a transthoracic echocardiographic examination within 1 month before the procedure. The left atrial diameter (LAD) was measured from two-dimensional images in the parasternal long-axis view. The left ventricular ejection fraction (LVEF) was calculated using biplane Simpson’s method.

### Coronary vasospasm diagnosis

A diagnosis of coronary spasms was determined when a spastic coronary artery without any significant coronary stenosis or air embolization was confirmed by coronary angiography (CAG) during ST-T elevation or just after an improvement in the ST-T elevation with an infusion of nitroglycerin [8]. When the coronary angiography could not be performed for some reason, such as avoiding the use of contrast medium in patients with severe bronchial asthma or when stabilizing the vital signs was prioritized, we conducted coronary computed tomography (CT) after the RFCA procedure, in which pretreatment with steroids was performed if necessary. Cardiac tamponade was excluded on emergent echocardiography.

### Statistical analysis

The measurements are presented as the mean value ± standard deviation. For the continuous and categorical variables, the differences between groups were compared using the Student *t* test and Fisher exact test, respectively. A *P* value < 0.05 was considered statistically significant. All statistical analyses were performed with SPSS version 25.0 J software for Windows (SPSS Inc., Chicago, IL, USA).

## Results

**Fig. 1:**
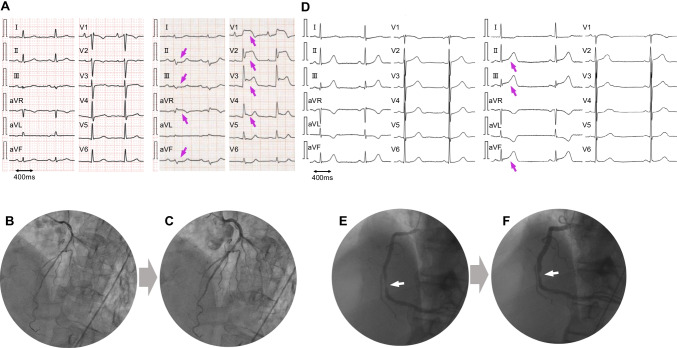
Electrocardiograms and coronary angiograms during the ST-T elevation. **A–C** The electrocardiogram (ECG) (**A**) demonstrates ST-T elevation in the precordial leads in case 1 (69 years old, male). The coronary angiography before (**B**) and after an intra-coronary nitroglycerin injection (**C**) reveals coronary artery spasms. **D**–**F** An ECG in case 2 showing ST-T elevation in leads II, III, and aVF (**D**). A 90% stenosis of the right coronary artery is shown (**E**); however, an intra-coronary nitroglycerin injection relieved the stenosis (**F**)

After the exclusion criteria were applied, a total of 2913 patients were analyzed. Among them, 9 patients (0.31%, mean 66 ± 10 years, 7 males) had transient ST-T elevation fulfilling the criteria of CAS during the perioperative period of the ablation procedure. Table 1 shows the clinical characteristics of the patients with CAS. ST-T elevation occurred directly after the transseptal puncture in 7 patients, just after the sheath was pulled out of the left atrium after the PV isolation in 1, and 2 h after the ablation procedure in 1. Eight out of 9 patients had ST-T elevation in the inferior leads, while concomitant bradyarrhythmias and hypotension were observed in 4 and 5 patients, respectively. Urgent coronary angiography was carried out in 8 patients and no significant fixed stenosis was revealed in any of them (Fig. 1). Of the 8 patients who underwent an emergency CAG, 6 had definite CAS. One of the patients with definite CAS did not receive nitroglycerin because their ST-T elevation returned to baseline relatively early. In one of the eight patients who underwent an emergency CAG, intracoronary infusion of nitroglycerin was performed before a left CAG, and the ST-T levels rapidly improved with the medication. A subsequent right CAG showed no CAS. In the other case, the ST-T elevation improved spontaneously within about 4 min, and a CAG performed immediately afterwards showed no air embolization and no significant stenosis or slow flow. In one patient who did not undergo an urgent CAG, their ST-T level promptly improved to normal within 3 min after intravenous nitroglycerin. No significant stenosis was revealed by coronary CT angiography after the AF ablation in that patient. During a mean follow-up of 2.2 ± 3.0 years after the procedure, no coronary events have occurred in any of the 9 patients.

**Table 1:** Patients with coronary artery spasms

Case	Age (years)	Sex	Combined disease	Smoking; Brinkman index	Beta-blocker Y/N	Calcium channel	AF type	CHADS_2_ score	CHA_2_DS_2_-VASc score	LAD (mm)	When spasms were observed	Localization of ST-elevation in the 12-lead ECG	Bradycardia Y/N	Hypotension Y/N	Anesthesia
1	69	M	None	2880	Y	N	Per AF	0	1	37	2 h after CA	V1-5, aVR	N	Y	Midazolam + dexmedetomidine
2	65	M	HTN	900	Y	Y	PAF	1	2	38	When sheath was pulled out after	II, III, aVF	Y	Y	Propofol
3	41	M	None	60	N	N	PAF	0	0	32	After Brockenbrough	II, III, aVF	N	N	Propofol
4	74	M	DM, DL	600	N	N	PAF	1	2	32	After Brockenbrough	II, III, aVF	N	N	Midazolam + dexmedetomidine
5	74	F	HTN, DL	540	N	N	PAF	1	3	45	After Brockenbrough	II, III, aVF	Y	Y	Midazolam + dexmedetomidine
6	72	M	None	780	N	N	PAF	0	1	28	After Brockenbrough	II, III, aVF	N	N	Midazolam + dexmedetomidine
7	66	F	HTN, DL, BA	0	Y	Y	PAF	1	3	28	After Brockenbrough	II, III, aVF	N	Y	Midazolam + dexmedetomidine
8	66	M	HTN, DL	540	N	Y	Per AF 2nd	1	2	39	After Brockenbrough	II, III, aVF	Y	Y	Midazolam + dexmedetomidine
9	63	M	None	900	N	N	Per AF	0	0	42	After Brockenbrough	II, III, aVF	Y	N	Midazolam + dexmedetomidine

*AF* atrial fibrillation, *BA* bronchial asthma, *CA*, catheter ablation, *Dex* dexmedetomidine, *DL* dyslipidemia, *DM* diabetes mellitus, *HTN* hypertension, *LAD* left atrial diameter, *PAF* paroxysmal atrial fibrillation, *Per AF* persistent atrial fibrillation

### Correlation between the CAS and other variables

The patients with CAS had a higher frequency of a smoking habit (89% vs. 53%; *P* = 0.04), smaller LAD (36 ± 6 vs. 40 ± 7; *P* = 0.07), and lower CHADS_2_ score (0.6 ± 0.5 vs. 1.3 ± 1.1; *P* = 0.004) than the patients without CAS. There were no significant differences in the other parameters including the use of dexmedetomidine (78% vs. 73%; *P* = 1.00), beta-blockers (33% vs. 47%; *P* = 0.51), and calcium channel blockers (33% vs. 31%; *P* = 1.00), concomitant dyslipidemia (44% vs. 45%; *P* = 1.00), and a previous AF ablation procedure (11% vs. 25%; *P* = 0.47) between the patients with and without CAS (Table 2). Also, no difference was seen in the use of an RF needle (66% vs. 52%; *P* = 0.51).

**Table 2:** Comparison of the parameters between the patients with and without coronary artery spasms

	CAS (*n* = 9)	no-CAS (*n* = 2904)	*P* value
Age (years)	66 ± 10	65 ± 11	0.77
Male (%)	78	72	1.00
Body mass index (kg/m^2^)	25 ± 3	24 ± 4	0.80
Paroxysmal AF (%)	67	66	1.00
Non-paroxysmal AF (%)	33	34	1.00
1st session (%)	89	75	0.47
Heart failure (%)	0	17	0.37
Hypertension (%)	33	59	0.17
Diabetes mellitus (%)	22	15	0.63
Stroke (%)	0	9	1.00
Dyslipidemia (%)	44	45	1.00
CHADS_2_ score	0.6 ± 0.5	1.3 ± 1.1	0.004
CHA_2_DS_2_-VASc score	1.6 ± 1.1	2.2 ± 1.5	0.14
Dexmedetomidine use (%)	78	73	1.00
RF needle use (%)	66	52	0.51
Beta-blocker use (%)	33	47	0.51
Calcium channel blocker use (%)	33	31	1.00
Smoking habit (%)	89	53	0.04
Smoking; Brinkman index	800 ± 845	354 ± 595	0.15
Left atrial diameter (mm)	36 ± 6	40 ± 7	0.07
Left ventricular ejection fraction (%)	65 ± 7	65 ± 11	0.86
Creatinine (mg/dL)	1.0 ± 0.2	1.0 ± 0.7	0.99
CCr (mL/min)	74 ± 19	79 ± 34	0.43

Values are given as the mean ± standard deviation or as the number (%)

*AF* atrial fibrillation, *CAS* coronary artery spasm, *CCr* creatinine clearance

## Discussion

This study was performed in a Japanese population with a relatively large number of patients. The patients with CAS during the perioperative period of the ablation procedure had a higher frequency of a smoking habit, smaller left atrium (LA), and lower CHADS_2_ scores than those without, while no differences were seen in the use of beta blockers or dexmedetomidine.

Vascular smooth muscle hyper-reactivity is shown to be the primary pathophysiological mechanism responsible for CAS, with endothelial dysfunction also potentially contributing to them [9]. Spontaneous CAS attacks associated with ST-segment changes usually occur at rest or during the night to early morning, and smoking is shown to be a significant risk factor for developing CAS [10].

The mechanism of CAS and risk factors of the perioperative ablation procedure have not been well established. According to several previous reports, the proposed mechanisms of CAS related to the ablation procedure include the direct thermal effects on the coronary artery [11], indirect effects via cryoenergy-induced blood cooling [5], and an autonomic nervous system imbalance caused by the affected ganglionated plexus through thermal or cooling injury [12–15]. However, in our study, 7 out of 9 patients had CAS directly after the transseptal puncture before the RFCA, suggesting no effect of the thermal energy. Nakamura et al. reported the CAS related to the AF ablation procedure [16]. CAS associated with AF ablation occurred in 0.19% (42 of 22,232) and CAS occurred before the ablation in 21%. The occurrence rate of CAS was similar in our study. The low incidence of CAS during and after the treatment in this study might have been related to the fact that there was little treatment with cryoablation, which is believed to be more likely to cause CAS associated with cooling, and that CFAE ablation, which might affect the autonomic nervous system, was not performed in our hospital. The novelty of this study was that we compared the patients with and without CAS.

The atrial septum is concentrated with a high density of parasympathetic fibers, which preferentially innervate the right coronary artery leaving it vulnerable to cholinergic vasospasms [17]. Passage of the catheter through these high-density nerve complexes of the atrial septum during transseptal catheterization might irritate or damage the plexus, then inducing a Bezold-Jarisch like reflex. When ST-T elevation occurred, 4 and 5 patients had bradycardia and hypotension, respectively, which was consistent with the response of a Bezold-Jarisch-like vasovagal response. These symptoms were likely to be mediated by the mechanical effects of the puncture on the vagal network located close to the Brockenbrough site. In our study, the patients with CAS tended to have a smaller LAD than those without. The nerve fiber distribution in the smaller atria might be vulnerable to a reflex activation during the transseptal puncture. One patient experienced CAS when the catheter sheath was pulled from the left to the right atrium after the pulmonary vein isolation. The mechanism of CAS in that patient might have been the same mechanical stimulation of the atrial septum.

Another report indicated that the cause of transient ST elevation was an air embolism. Kuwahara et al. reported that serious air embolisms occurred in patients with long apneic spells under sedation during AF ablation [18]. Air emboli can be introduced from the transseptal sheaths and migrate into the right coronary artery because the right coronary cusp is positioned at the superior aspect of the heart when the patients are in the supine position. In our study, however, the coronary angiography during the ST-T elevation did not demonstrate any air embolisms in any cases. One of the possible reasons was that many doctors have become very careful about preventing an air embolization, and have taken measures to prevent snoring during the procedures and pulling in air during the sheath insertion and removal after that meaningful report [18] from the Japanese institutions.

There have been some reports [13, 19] that dexmedetomidine, an α-2 adrenergic receptor stimulant, administered with a loading dose might be highly likely to be responsible for CAS. It has been shown that α-2 adrenoreceptor-mediated vasoconstriction can affect the coronary circulation, especially in the presence of atherosclerosis and endothelial dysfunction [20]. However, in this study, no significant difference was found in the use of dexmedetomidine between the patients with and without CAS. There was also no difference in the use of beta-blockers between the patients with or without CAS. Beta-blockers are thought to have an aggravating effect on CAS due to the inhibition of beta-adrenergic receptor-mediated coronary vasodilation, unmasking the alpha-adrenergic tone, and increasing the vascular permeability to calcium [21]. Although the use of dexmedetomidine or beta-blockers is a risk factor causing CAS during the ablation procedure, these agents might affect the occurrence of CAS more likely in patients with structural disorders or other conditions.

CAS during regional or extradural anesthesia in patients undergoing non-cardiac surgeries have been described in some case reports and may be related to an inadequate analgesia level [22] and sympathetic blockade [23, 24]. A sympathetic blockade reduces the peripheral resistance, and the reflex sympathetic activity induces a compensatory vasoconstriction, which might cause CAS. In a study of 77,745 consecutive patients who underwent a non-cardiac surgery, 18 had CAS [25]. The Revised Cardiac Risk Index, which is a predictive measure of the risk of a cardiac event during the perioperative period [26], was low (0.5 ± 0.6) in the CAS patients. In the present study, the patients with CAS had lower CHADS_2_ scores than those without. The possible risk of CAS during the ablation session might be unrelated to the cardiovascular risk factors.

### Limitations of the study

This study had several limitations. First, the possibility of air embolisms or small plaque as a cause of CAS was not completely ruled out. In 8 of 9 patients with ST-T elevation, however, no recognizable embolus or clot was observed, when we performed coronary angiography. Although most patients with ST-T elevation improved immediately after the nitroglycerin infusion and were likely to have CAS, the possibility that they also improve with an air embolization could not be ruled out. All 9 patients with ST-T elevation did not have sleep apnea and the respiratory condition was stable during the procedure. Whether the ST-T elevation was due to micro-plaque or not could not be denied, the coronary risk factors, however, were lower except for a smoking habit in patients with ST-T elevation than in those without. Second, this was a study in a single Japanese center, which limited the generalizability of our results. Some treatments, including cryoablation, were limited. A larger international multicenter study is warranted to confirm the results. Third, ST-T elevation with CAS may occur without any hypotension or bradycardia. Therefore, there might be cases in whom the ST-T elevation was missed, although the surface ECGs were continuously monitored throughout the ablation in order not to overlook any cardiac abnormalities. Fourth, in the cases in this study, the CAS may have been underdiagnosed before the ablation session. All 9 cases, however, had neither a history of chest pain prior to nor episodes of ST-T segment changes detected on Holter ECG monitoring performed before the procedure. During a follow-up of 26 months, there have been no signs of CAS in any of the 9 patients.

## Conclusions

Among the 2913 patients that underwent AF ablation, CAS occurred in 9 patients (0.31%). Compared to the patients without CAS, those with CAS had a higher frequency of a history of smoking and a smaller left atrium. Although the incidence of CAS was rare, it should be kept in mind as a serious complication of AF ablation. CAS predominantly occur after the transseptal puncture but may occur a few hours after the procedure. As the number of AF ablation procedures increases, that of CAS patients during the perioperative period of the ablation procedure may also rise. Therefore, careful monitoring of the surface ECGs is necessary during AF ablation to quickly and properly address this rare but potentially life-threatening complication.

## Data Availability

The data that support the findings of this study are available from the corresponding author, Yuhi Fujimoto, upon reasonable request.
