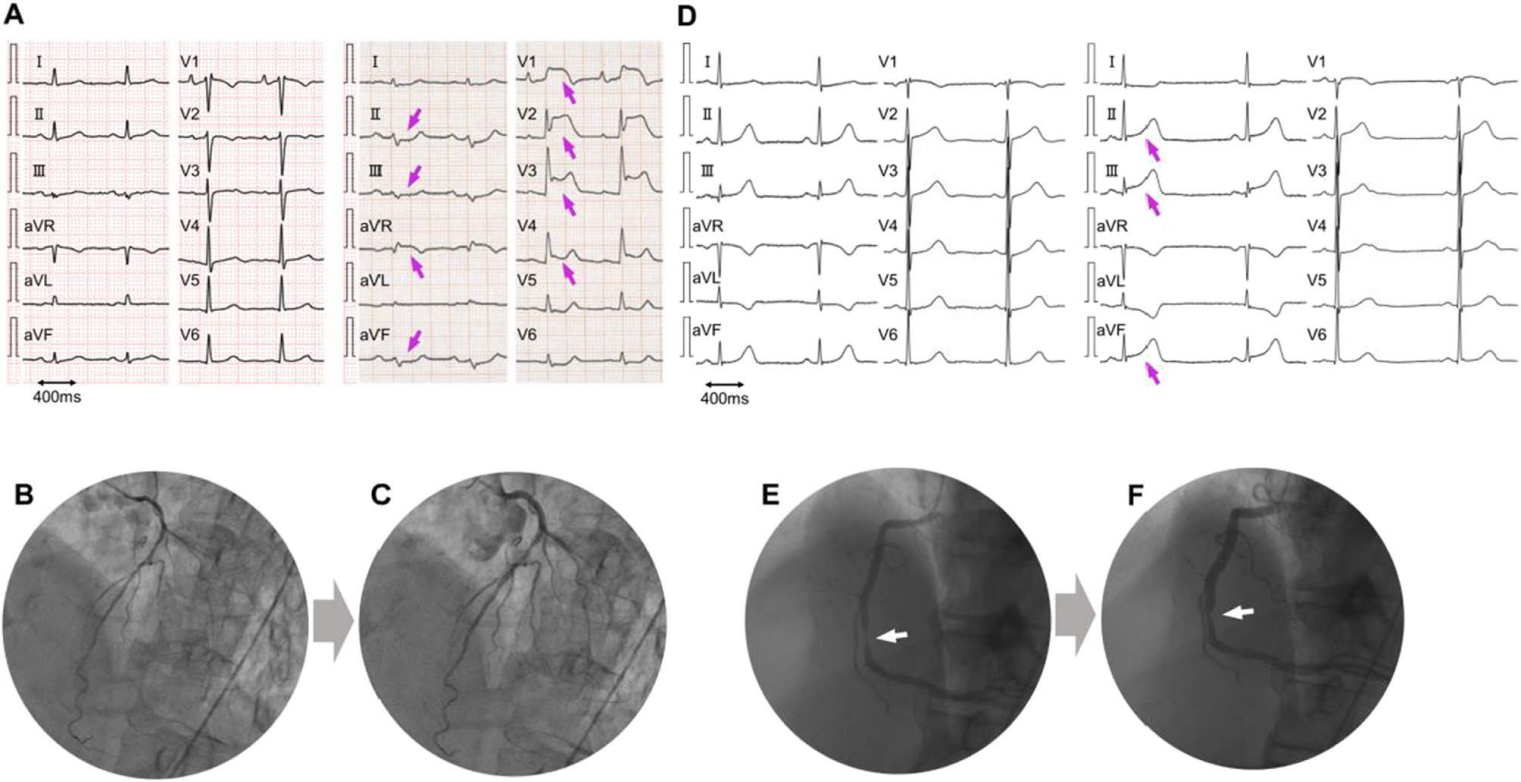# Correction to: Perioperative coronary artery spasms in patients undergoing catheter ablation of atrial fibrillation

**DOI:** 10.1007/s10840-022-01161-9

**Published:** 2022-02-24

**Authors:** Masato Hachisuka, Yuhi Fujimoto, Eiichiro Oka, Hiroshi Hayashi, Teppei Yamamoto, Hiroshige Murata, Kenji Yodogawa, Yu-ki Iwasaki, Meiso Hayashi, Yasushi Miyauchi, Wataru Shimizu

**Affiliations:** 1 Department of Cardiovascular Medicine, Nippon Medical School, 1-1-5 Sendagi, Bunkyo-ku, Tokyo, 113-8603 Japan; 2 Mabori Medical Clinic, Yokosuka, Japan

**Correction to: Journal of Interventional Cardiac Electrophysiology**

**https://doi.org/10.1007/s10840-021-01089-6**

In this article a scale has been added to the right side of the precordial leads in Figure 1A, and it just covers the ECG.; the figure should have appeared as shown below.

The original article has been corrected.